# Avian blood parasite infection during the non-breeding season: an overlooked issue in declining populations?

**DOI:** 10.1186/1472-6785-13-30

**Published:** 2013-09-06

**Authors:** Jenny C Dunn, Simon J Goodman, Tim G Benton, Keith C Hamer

**Affiliations:** 1 School of Biology, Irene Manton Building, University of Leeds, Leeds LS2 9JT, UK; 2 Centre for Conservation Science, Royal Society for the Protection of Birds, The Lodge, Sandy, Bedfordshire SG19 2DL, UK

**Keywords:** Sub-clinical disease, Parasite ecology, Farmland birds, Declining populations, *Emberiza citrinella*, Yellowhammer, *Haemoproteus*

## Abstract

**Background:**

Pathogens and parasites can have major impacts on host population dynamics, both through direct mortality and via indirect effects. Both types of effect may be stronger in species whose populations are already under pressure. We investigated the potential for blood parasites to impact upon their hosts at the immunological, physiological and population level during the non-breeding season using a declining population of yellowhammers *Emberiza citrinella* as a model.

**Results:**

Yellowhammers infected by *Haemoproteus* spp. showed both a reduced heterophil to lymphocyte (H:L) ratio, and an elevated standardised white blood cell (WBC) count compared to uninfected birds, indicating an immunological response to infection. Infected birds had shorter wings during the first winter of sampling but not during the second, colder, winter; survival analysis of 321 birds sampled across four winters indicated that increased wing length conferred a survival advantage.

**Conclusions:**

We suggest that the potential impacts of blood parasite infections on over-wintering birds may have been underestimated. Further research should consider the potential impacts of sub-clinical parasite infections on the dynamics of vulnerable populations, and we suggest using declining populations as model systems within which to investigate these relationships as well as examining interactions between sub-clinical disease and other environmental stressors.

**JEL Code:**

Q5

## Background

Pathogens and parasites can have major direct impacts on host population dynamics through increases in mortality and morbidity [1,2], sometimes leading to host extinction [3-5]. However, they can also have more subtle effects on host physiology [6,7], behaviour [8] and ecology [9], which can also affect host survival and population dynamics [6].

The impacts of pathogens on their hosts are dependent upon the ecology, behaviour and life history of both host [10] and parasite [11] species. Environmental factors such as variation in ambient temperature can increase or decrease host condition and therefore susceptibility, as well as parasite behaviour and virulence, leading to a critical influence on the outcome of host-parasite interactions, even over relatively small temperature ranges [12]. Environmental stress, such as that caused by extreme temperature events [13], reduced food availability [14], exposure to pesticides [15] or anthropogenic disturbance [16] may thus amplify the costs of parasitism [17] through impacts on the costs of an immune response [18] through increased energy demands [19].

Any impacts of parasitic infection may be particularly important in declining populations, which are often under additional pressure from factors such as loss of habitat and food [20-22]. Multiple stress-inducing factors can have synergistic effects [23-25], with both physiological [23] and ecological consequences [24,26]. Declining populations may thus be at greater risk than stable populations from the sub-lethal effects of pathogens and parasites.

Previous studies of avian blood parasites have mainly investigated impacts of parasitism during the breeding season, when environmental stress tends to be relatively low but hormones may reduce the efficacy of the host immune system leading to relapses of infection [27,28]. These studies have shown consistent negative impacts of blood parasite infection on reproductive performance across all stages of breeding [29-32], as well as associations with physiological variables such as feather length and bill colour [9,33] which are likely to be determined outside the breeding period.

Allander and Sundberg [34] suggested that the possible costs of parasites outside the breeding season required further study. Despite this, the potential impacts of blood parasites during the non-breeding season have seldom been investigated. Two exceptions are recent work by Cosgrove et al. [35], who found *Plasmodium* spp. to be absent from a population of blue tits *Cyanistes caeruleus* during the winter months, and by Barnard et al. [36], who found haematozoa to be present in wintering populations of the declining rusty blackbird *Euphagus carolinus* and suggest a non-seasonal relapse of infections among overwintering birds. Martínez-de la Puente et al. [37] found a reduction in survival between infected and medicated blue tits, suggesting differential mortality outside the breeding season. We suggest that, in temperate regions, the impacts of parasites and pathogens may be just as severe during the winter months, when food is scarce and temperatures are lower than during the breeding season, leading to higher energetic trade-offs between immune function and thermostatic maintenance e.g. [18,19].

Here, we carry out an exploratory study into the potential for blood parasites to impact their avian hosts outside the breeding season. We use a population of yellowhammers *Emberiza citrinella*, a declining farmland specialist of conservation concern in the UK [38], infected with blood parasites of the genus *Haemoproteus. Haemoproteus* parasites are generally transmitted during the breeding season by vectors of the Families *Ceratopogonidae* and *Hippoboscidae*[39], and parasite intensity in passerine hosts peaks during the breeding season e.g. [40]. Hosts usually recover from infection but Allander & Sundberg [34] point out that *Haemoproteus* infections are likely to occur in their hosts long after the breeding season. We examine associations with sub-clinical disease at the immunological and physiological levels, and we use a physiological trait linked with infection to examine potential population-level implications of infection. Whilst our data are correlative, our main aim here is to highlight the potential for such mechanisms to act outside of the breeding season (when parasites are generally thought to have detrimental effects), and to suggest directions and priorities for future research.

## Results

Of the 225 samples from 203 birds that were screened for the presence of haematozoa, 47% of samples were positive for *Haemoproteus* infection. We found two strains of *Haemoproteus* in our population [41], but as we only sequenced a subset of positive samples, we grouped these two strains together for further analysis. Infection intensity is described elsewhere in more detail [41], but mean infection intensity in a random sub-sample of birds confirmed as infected through PCR (n = 44) was 0.38 parasites per 10,000 erythrocytes (range 0 – 7.03 parasites per 10,000 erythrocytes). Year-specific data summaries for all variables are provided in Additional file 1.

### Immunological impacts of blood parasites

Measures of both chronic stress and immune system activity (H:L ratio and standardised WBC count respectively) were influenced by parasite infection status (Table 1). Controlling for lymphocyte numbers, infected birds had 35% lower heterophil counts on average than uninfected birds (infected: 0.217 ± 0.025; uninfected: 0.335 ± 0.031), resulting in a reduced H:L ratio. Similarly, controlling for WBC numbers, infected birds had fewer erythrocytes compared to uninfected birds, resulting in a 60% higher mean standardised WBC count (infected: 0.715 ± 0.081; uninfected: 0.449 ± 0.055 white cells per 1000 erythrocyes).

**Table 1:** **Results of general linear models determining whether H:L ratio or standardised WBC count are influenced by *Haemoproteus *infection**

	**H:L ratio**					**WBC index**				
**Variable**	**df**	**F**	**p**	**Estimate**	**SE**	**df**	**F**	**p**	**Estimate**	**SE**
Number of lymphocytes	1, 70	330.88	<0.001	-0.029	0.002	NA	NA	NA		
Number of RBCs per slide view	NA	NA	NA			1, 66	1.521	0.222	-0.006	0.004
Parasite infection status (Infected)	1, 69	10.547	0.002	-0.235	0.067	1, 65	4.439	0.039	-0.372	0.173
Time of day	1, 64	0.147	0.703			1, 63	6.829	0.011	-0.095	0.042
Age (Juvenile)	1, 65	1.027	0.315			1, 61	0.404	0.528		
Sex (Male)	1, 66	1.195	0.278			1, 62	3.550	0.064	0.334	0.180
Year (2008)	1, 67	2.034	0.158			1, 64	4.556	0.037	-0.319	0.174
Month (December)	1, 68	3.856	0.026	-0.053	0.088	1, 60	0.004	0.947		
Month (February)				-0.232	0.096					

For significant terms, parameter estimates with SE are presented; for non-significant main effects, statistics follow reinsertion of the term into the minimum adequate model (MAM) and subsequent model comparison. Parameter estimates for two level factors are for the stated factor compared to the other factor; estimates for month are for the stated month when compared to April. ‘NA’ indicates that this term was not included in the model.

### Morphological associations with parasitism

**Figure 1:**
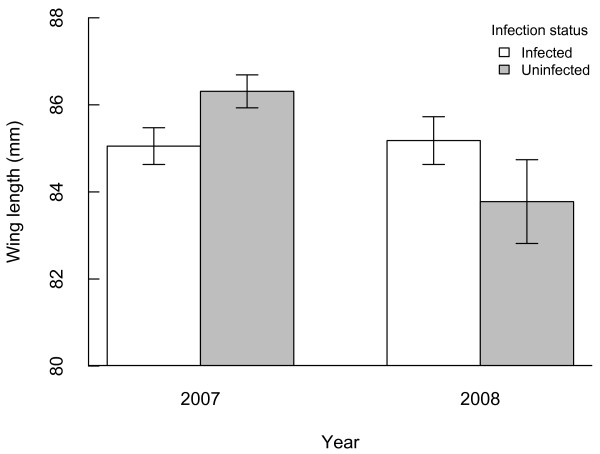
**The effect of parasite infection on wing lengths of yellowhammers in winter 2007 and 2008.** Bars show mean ± 1 SE.

Wing length was associated with both an interaction between age and sex (as described in Dunn and Wright [42]), and an interaction between *Haemoproteus* infection status and year (Table 2). Post hoc analyses indicated that in the winter of 2007/08, birds infected with *Haemoproteus* had shorter wings than uninfected birds (t_1, 139_ = 2.069, p = 0.04) but in the winter of 2008/09, this was not the case (t_1, 52_ = −1.235, p = 0.222; Figure 1). When the lowest maximum temperature prior to the sampling date during each winter was substituted into the minimum adequate model (Table 2) in place of year, the model explained the same amount of variation as that containing year (Model containing year: adjusted R^2^ = 0.579; Model containing lowest maximum temperature: adjusted R^2^ = 0.581), with mean population wing length decreasing as lowest maximum temperature increases. No association with infection status was found for either head-beak length (F_1_ = 0.31, p = 0.58; infected: 30.21 ± 0.07 mm; uninfected: 30.13 ± 0.06 mm; see Additional file 2 for full model results) or tarsus length (F_1_ = 0.68, p = 0.41; infected: 18.30 ± 0.10 mm; uninfected: 18.24 ± 0.09 mm; see Additional file 2 for full model results).

**Table 2:** **Results from a GLM to determine whether infection by *Haemoproteus*, or any interactions therewith, are associated with wing length**

**Variable**	**df**	**F**	**p**	**Estimate**	**SE**
Sex (Male)	1, 200	10.236	<0.001	5.231	0.511
Age (Juvenile)	1, 200	−5.214	<0.001	−2.580	0.495
*Haemoproteus* infection (uninfected)	1, 200	2.057	0.041	0.807	0.393
Year (2008)	1, 200	0.904	0.367	0.434	0.480
Sex x Age	1, 200	−2.184	0.030	−1.455	0.666
Month	1	0.545	0.742		
*Haemoproteus* infection x Year	1, 200	−2.143	0.033	−1.638	0.764
*Haemoproteus* infection x Sex	1	0.970	0.330		
*Haemoproteus* infection x Age	1	1.420	0.220		

For significant terms, parameter estimates with SE are presented; for non-significant terms, statistics follow reinsertion of the term into the minimum adequate model (MAM) and subsequent model comparison.

### Survival analysis

Using the most parsimonious recapture model (Table 3), survival models were estimated (Table 4). The ∆QAICc between the top three models was <1, so we used model averaging to produce parameter estimates (Table 5). Survival was positively affected by wing length and sex (males survived better than females), and recapture probability decreased with time, although not linearly (Table 5).

**Table 3:** Summary of the candidate models predicting the probability of recapture (p)

**Model**	**Φ**	**p**	**npar**	**QAICc**	**∆QAICc**	**Model weight**
1	1	Time	4	164.52	0.000	0.555
2	1	Constant	2	167.10	2.572	0.153
3	1	Wing length	3	168.34	3.821	0.082
4	1	Sex	3	168.42	3.895	0.079
5	1	Age	3	169.10	4.578	0.056
6	1	Age + Wing length	4	170.26	5.738	0.031
7	1	Sex + Wing length	4	170.29	5.764	0.031
8	1	Age + Sex + Wing length	5	172.25	7.723	0.012

Φ (the probability of survival) remains as a constant in this model; npar shows the number of parameters; QAICc is the adjusted AIC value following correction for overdipersion, and ∆QAICc shows the difference between the specified model and the ‘best’ model. Models with a ∆QAICc < 2 are considered not to differ from the best model.

**Table 4:** Summary of the candidate models predicting the probability of survival (Φ)

**Model**	**Φ**	**p**	**npar**	**QAICc**	**∆QAICc**	**Model weight**
1	Constant	Time	4	163.68	0.000	0.273
2	Wing length	Time	5	163.86	0.185	0.249
3	Sex	Time	5	164.67	0.995	0.166
4	Age	Time	5	165.71	2.034	0.099
5	Age + Wing length	Time	6	165.85	2.176	0.092
6	Sex + Wing length	Time	6	165.92	2.243	0.089
7	Age + Sex + Wing length	Time	7	167.93	4.257	0.032

The model with Time alone was found to be the best model for predicting the probability of recapture (p), and thus p remains as a constant in this model; npar shows the number of parameters; QAICc is the adjusted AIC value following correction for overdipersion, and ∆QAICc shows the difference between the specified model and the ‘best’ model. Models with a ∆QAICc < 2 are considered not to differ from the best model.

**Table 5:** Averaged model estimates predicting survival (Φ) and recapture (p) probabilities for yellowhammers

	**Estimate**	**SE**	**LCL**	**UCL**
Φ				
Wing length (75 mm)	0.434	0.058	0.374	0.595
Wing length (95 mm)	0.686	0.058	0.447	0.722
Sex Male	0.632	0.170	0.322	0.920
Sex Female	0.588	0.141	0.263	0.767
p				
Time 2 (2006/07)	0.206	0.069	0.102	0.372
Time 3 (2007/08)	0.160	0.108	0.038	0.478
Time 4 (2008/09)	0.150	0.204	0.008	0.801

## Discussion

Understanding the drivers of population dynamics is a major challenge in population biology, particularly when populations are declining and require mitigation strategies to prevent extinction. Many European farmland birds have declined following farmland intensification and this has been hypothesised as being due to combinations of loss of habitat, shortages of summer and winter food, or a multiplicity of causes [20-22,43]. Here we add a further potential cause as we show, for the first time, high parasite prevalence in winter, a season associated with significant mortality. This is coupled with evidence suggestive of immunological challenge and a physiological response that may potentially impact upon survival, and we suggest that declining populations may make good systems within which to examine this previously overlooked period in host-parasite ecology. Whilst results here are purely correlative, we discuss the potential implications of our data that warrant further, experimental, work.

The high prevalence of infection found in our population suggests either a high incidence of chronic infections, or stress-induced relapse of existing infections. The vectors of blood parasites tend to be dormant during the winter months, considerably reducing the transmission of new infections [35]. For *Haemoproteus* parasites, microscopy and PCR techniques tend to provide similar prevalence estimates [44,45], although Fallon & Ricklefs [46] found 25-35% of PCR-detected *Haemoproteus* infections to be undetected through examination of blood smears. Infection intensities within our population are relatively low when compared to other passerine-*Haemoproteus* spp. systems (e.g. 40–790 parasites per 10,000 RBCs [47]; means of 6.4 and 12.1 parasites per 10,000 RBCs in the West Indies and Missouri Ozarks respectively [46]; 90 parasites per 10,000 RBCs [48]) during the summer months, possibly suggesting that our findings represent the chronic stages of infection, rather than relapses. Unfortunately infection intensity data from our study species during the breeding season is, to our knowledge, only presented in Allander and Sundberg [34] but this paper measures parasites per 100 microscope fields and does not standardise measurements by erythrocyte abundance, so we are unable to make direct comparisons, although these data from our population would be valuable in assessing the relative effects of parasitism between the breeding and non-breeding periods. However, as the non-infective asexual stages of *Haemoproteus* reside in the tissues and only infective gametocytes are present in the peripheral circulation [39,49], this suggests that our findings represent active infections (regardless of whether these are considered to be chronic infections, or relapses) and has further implications for the over-winter ecology of avian blood parasites in temperate species.

*Haemoproteus* infection was associated with both a reduced H:L ratio, and an increased WBC count. The H:L ratio is generally expected to increase in response to infection [50], whereas our data suggest the opposite. Similarly to our data however, other systems have also found a reduction in H:L ratio associated with *Haemoproteus* infection [51,52], and Galeotti and Sacchi [51] suggest that the reduction in H:L ratio is explained by the higher concentration of lymphocytes overall, triggered by an increase in immune system activity [53]. This is demonstrated in our data by the increased WBC count in infected individuals, and suggests a direct immune cost of *Haemoproteus* infection in this species. H:L ratio is also strongly positively correlated with corticosterone levels [50], but increases with increasing environmental stress, so these data do not support the idea that increased corticosterone, induced by food stress or poor weather, induces relapses of active parasite infection [36]. However, an increase in immune activity also carries costs [18]: for example, in the house sparrow *Passer domesticus*, *Haemoproteus* infection reduces immune response to a second immune challenge [54,55]. Increased immune activity leads to an increased short-term energy consumption which, even at the levels of a 5-15% increase in energy expenditure calculated by Hasselquist & Nilsson [19] may amplify the impacts of sub-clinical disease at times of food- or weather-related stress, or in populations already under environmental pressure.

We found year-dependent associations between parasite infection and reduced feather length, with no such relationship with skeletal measures of size, suggesting that under certain conditions, parasites can influence feather length through competition for host resources during moult [9,33], although we acknowledge that this relationship may be mediated by a third, unknown, factor such as body condition during moult. Whilst our parasite data are from the winter months, rather than from months when moult was occurring, the presence of parasites during winter suggests that they were also present during the preceding post-breeding moult (especially if our data represent chronic infections), as post-breeding tends to be the time when infection rates are highest in other systems [35]. In addition, parasite intensities between peak parasite intensity and chronic infections can be correlated [56] and novel infections between the end of moult and the winter months may be relatively unlikely due to cessation of vector activity, although further work would be required to confirm this. A larger dataset of repeat within-individual data between years would be of great value in elucidating whether this association with wing length occurs at an individual level in relation to infection intensity.

The replacement of year with lowest maximum temperature explained a similar amount of variation in the statistical model, suggesting that temperature may underlie the relationship between parasitism, wing length and year. Parasite prevalence was higher during the second year of the study, coinciding with fewer birds sampled despite similar sampling effort [41], suggesting fewer birds in the population. This is unlikely to be due to birds moving out of the study area during the second year, as yellowhammers in the UK show very low dispersal [57] and our population showed a relatively high winter-summer fidelity as estimated through colour-ring re-sightings (Dunn, J. C., Goodman, S. J., Benton, T. G. & Hamer, K. C., unpublished observations). Instead, this may have been due to an early cold spell during autumn 2008 (the second year of the study [58]), leading to an increase in energy requirements and consequently food requirements, thus resulting in increased over-winter mortality. Given the association between infection and wing length was found only during the first year of sampling when the weather was mild and more birds were caught, and that our survival analysis indicated that birds with longer wings had an enhanced survival probability, it seems plausible that those birds susceptible to physiological effects of parasitism may be more likely to succumb to food stress during a cold winter such as that of the second year of the study and thus be removed from the population. However, this suggestion is speculative and requires further investigation, preferably by inclusion of blood parasite intensity data in the survival analysis.

Farmland birds more generally are under pressure from reduced nesting habitat availability, reduced quality of available nesting habitat, and reductions in both summer and winter food availability [20-22,43]. Here, we suggest that parasites may also play a significant role in the ecology of declining populations, potentially by interacting with other environmental stressors. These interactions have the potential to occur both within and outside the breeding season, and we suggest that declining populations, especially those such as ours that remain resident outside the breeding season, would make excellent study systems for the examination of such relationships within and between seasons.

## Conclusions

Whilst our data are correlative and thus our results are speculative in terms of causality, we show associations between parasite infection and immune function, and between parasite infection and a physiological trait that we show to be of ecological significance. Our data suggest that the impacts of parasites outside the breeding season may be more important than previously thought, especially in species already under stress due to other ecological factors, such as food stress. Consequently, we also suggest that future experimental research on the ecological impacts of parasitism should examine potential impacts outside the breeding season, and should focus on populations already under pressure due to other ecological factors where these effects are likely to be more pronounced. Our results further suggest that considering sub-clinical disease may be critical when examining the conservation of declining populations.

## Methods

### Study population and blood sampling

Work was carried out within a population of yellowhammers near Tadcaster, North Yorkshire, UK (lat. 53˚ 53′N, long. 1˚ 15′W). Birds were caught in static mist nets and whoosh nets [59] at an established supplementary feeding site baited with wheat and weed seeds, within an experimental agroforestry block surrounded by arable farmland. 203 birds were caught on 30 sampling occasions between November and April during the winters of 2007/08 and 2008/09. The mean 1st egg date for yellowhammers in the UK is 29th May and the earliest broods are initiated in early May [60] so there was minimal overlap between our sampling period and the start of the breeding season. Furthermore, if physiological and behavioural changes such as that found to induce breeding-season relapses were present towards the end of our sampling period, we would have seen a higher prevalence in April samples compared to those from previous months, which was not the case (April prevalence: 48% (n = 33); December – March prevalence: 46% (n = 192)). Sixteen birds were caught and sampled on two occasions within this period and three birds were caught and sampled on three separate occasions more than two months apart. Blood was taken through venipuncture of the brachial vein and a blood smear created. Additional blood was stored with EDTA as an anti-coagulant and frozen prior to DNA extraction.

### Morphometrics

Birds were aged and sexed according to plumage variation [42,61]. Morphometrics were taken as described by Dunn and Wright [42]: wing length was used as a measure of feather growth, and head-beak length and tarsus length as measures of body size. Measurements of wing length were taken using a slotted metal rule (± 1 mm) using standard methodology [59]; other measurements were taken using digital callipers (± 0.1 mm).

### DNA extraction and detection of blood parasites

DNA was extracted from 30 of whole blood using a standard phenol-chloroform extraction followed by ethanol precipitation [62]. Successful DNA extraction was confirmed by using a Nanodrop ND-1000 Spectrophotometer (Nanodrop Technologies Inc., Wilmington, DE) and DNA was diluted to a working concentration of 25 – 100 ng/μl.

Blood parasite presence or absence (to establish parasite prevalence as the number of individuals with parasites present, divided by the total number of individuals sampled) was determined through PCR using established protocols. The presence of *Haemoproteus* spp. was established using primers HaemF and HaemR2 nested within HaemNF and HaemNR2 (this protocol also detects *Plasmodium* spp. but subsequent sequencing indicated that *Plasmodium* was absent from our sample [63]). All protocols were carried out in a working volume of 25 μl containing 50 – 200 ng template DNA, 1.25 mM dNTPs, 3 mM MgCl_2_, 0.4 μM of each primer, 1 x GoTaq Flexi Buffer (Promega, Madison, WI) and 1 U GoTaq Flexi (Promega, Madison, WI); a positive control of DNA from a bird for which sequence analysis had confirmed the presence of *Haemoproteus* parasites, and a negative control containing deionised water in place of DNA were included with each PCR reaction to ensure successful amplification and lack of contamination respectively.

The PCR protocol for first round reactions consisted of a denaturation step of 94°C for 3 minutes followed by 20 cycles of 94°C for 30 seconds, 50°C for 30 seconds and 72°C for 45 seconds, with a terminal extension step of 72°C for 10 minutes; the protocol for second round reactions contained 35 cycles but otherwise consisted of an identical thermal profile. PCR protocols were carried out on a GeneAmp PCR System 9700 (Applied Biosystems). As non-target DNA can be amplified with nested PCR methods [64] a subsample of positive samples were sequenced using an ABI sequencer at the Core Genomic Facility, Sheffield University, to confirm the identity of parasites.

### Immunological parameters

Blood smears were examined under an oil immersion x100 magnification lens to establish the intensity of infection (the number of parasites per 10,000 erythrocytes), the ratio of heterophils to lymphocytes (H:L ratio), and standardised white blood cell (WBC) count. H:L ratio and WBC count are indicative of increased levels of chronic stress [50] and immune system activity [53] respectively, and were measured to examine associations between indicators of immune function, and infection by *Haemoproteus*. From each smear, 100 WBCs were identified, and the frequencies of heterophils, eosinophils, basophils and lymphocytes were established by comparison with standard avian guidelines [65]. To examine overall WBC count, the approximate number of erythrocytes per slide view and the number of slide views required in order to view 100 WBCs was recorded to establish the ratio of WBCs to erythrocytes.

### Statistical analysis

Statistical analyses were carried out in R version 2.4.1 for Mac [66]. Model comparisons using AIC values were used throughout to determine whether terms significantly improved the fit of the model; those that didn’t were removed in a stepwise fashion until only those terms that improved the fit of the model at p < 0.1 remained. Following model simplification, each term was reinserted into the minimum adequate model (MAM) in turn and compared with the MAM using AIC comparisons to ensure lack of association with the response variable. Whilst stepwise deletion has been criticised in the literature [67], a recent study using real ecological data validated stepwise deletion as a method of model selection and concluded that it performed just as well as other methods of producing predictive models [68]. Furthermore, simplification of our statistical models through stepwise deletion made no difference to the terms considered important in influencing the response variables when compared to the maximal model.

Summary statistics are presented throughout as mean ± 1 SE, except where otherwise stated. Two data points per bird were available in only a very small number of cases where a bird was caught twice either within or between winters, so one point was selected at random for each bird to avoid pseudoreplication as the sample size of replicate points was too small to allow the use of mixed-effects models.

### Immunological impacts of blood parasites

To determine whether or not parasitism affected the H:L ratio, a general linear model (GLM) was constructed with heterophil number as the response variable and lymphocyte number included as a covariate: this modelled the relationship between heterophil number and other dependent variables, whilst controlling for lymphocyte number. A quasipoisson error structure was assumed. To determine whether or not parasite infection was associated with the standardised WBC count, the number of slide views required to find 100 WBCs was used as the response variable (as 100 WBCs were counted for each bird), with the mean number of RBCs per slide view as a covariate in a GLM with quasipoisson error distributions. In both models, age, sex, parasite status (infected or not), time of day and month were included as predictor variables, along with two way interactions between parasite status and each of age, sex and year, to determine whether any effects of parasitism on immunological variables varied between ages, sexes or years. To establish whether interactions with year were significant within each year, post hoc tests were carried out analysing the same model without the interaction term for each year separately.

### Morphological associations with blood parasite infection

To determine whether or not parasite infection showed any association with morphological variables, a linear model with Gaussian error distributions was constructed with wing length as the response variable. Model structure and predictor variables were identical to those constructed for estimating WBC index with the exception of the response variable; these models were repeated with head-beak length and tarsus length as response variables to determine whether any associations with parasitism were with size or with feather length. To examine significant interactions with year and examine where significant trends lay, post hoc analyses were carried out on data from each year separately, using t-tests. To determine if associations could be accounted for by differences in temperature within and between sampling years, lowest maximum daily temperature prior to sampling date (using data from the Met Office Hadley Centre Central England Temperature dataset [69]) was substituted into the model in place of year and the amount of variation explained by each model was inspected. Lowest maximum temperature was used because the maximum temperature determines whether the ground remains frozen during the day: frozen ground locks in seeds, thus limiting food availability for granivorous birds.

### Survival analysis

To estimate annual survival, the encounter histories of 321 individual colour-marked yellowhammers captured between December 2005 and May 2009 were analysed using Cormack-Jolly-Seber mark-recapture models [70]. Analysis was carried out in R version 2.8.0 for Windows using the package RMark [71] to construct models from program MARK [72]. This allows the calculation of survival probability and recapture probability separately [73], taking into account the fact that individuals may survive but not be recaptured. Encounter histories of birds were pooled into four time categories, comprising September – May during 2005/06, 2006/07, 2007/08 and 2008/09. As parasite status was available for birds captured during only the last two time periods, it was not included as a grouping variable; however, as wing length and parasite infection status were associated (see Results), we wanted to know whether or not wing length was an ecologically significant trait and thus we included wing length as a covariate. Birds were grouped according to sex and age (first winter or adult).

Firstly, the probability of recapture (*p*) was examined. The probability of survival (Φ) was set at a constant, and it was hypothesised that the probability of recapture might be dependent upon 1) Age, 2) Sex, 3) Time, and 4) Wing length. As age and sex both influence wing length [42], the combinations of age and wing length, sex and wing length, and age, sex and wing length were also used to construct models. The ĉ variance inflation factor was calculated and adjusted to control for over-dispersion of data, as is common within capture-recapture datasets [74]. This correction did not alter the model selection results. Models were selected on the basis of ∆QAICc values, with models differing in QAICc from the ‘best’ model by more than 2 considered to have a real difference from the best model [75]; thus, only models differing in QAICc from the best model by less than 2 were considered further.

The best recapture model was used to build survival models. The same factors as were thought to influence the probability of recapture were also considered the most likely to influence survival, with the exception of time. Thus, a further seven models were considered here. Models that did not differ from the best model (i.e. with a ∆QAICc of less than 2) were averaged to provide weighted estimates of the effects of important parameters on survival.

### Availability of supporting data

The sequences obtained during this study are available in GenBank, under accession numbers KF214775 and KF214776.

## Authors’ contributions

JCD, SJG, TGB and KCH conceived the study, planned data collection and wrote and revised the manuscript. JCD collected the field data, carried out laboratory analysis and performed the statistical analysis. All authors have read and approved the manuscript.

## Supplementary Material

Additional file 1 Summary data for variables split by year. Click here for file

Additional file 2 **Results from a GLM to determine whether infection by *Haemoproteus*, or any interactions therewith, is associated with either tarsus length or head-beak length.** For significant terms, parameter estimates with SE are presented (contrasts for Month are against the mean, contrasts for factors with two levels are for the level stated and compared to the other level); for non-significant main effects, statistics are following reinsertion of the term into the minimum adequate model (MAM) and subsequent model comparison. Click here for file
