# Supplementary material for: Avian blood parasite infection during the non-breeding season: an overlooked issue in declining populations?
Source: BMC Ecol. 2013 Sep 6;13:30. doi: 10.1186/1472-6785-13-30 (PMC3848531; doi:10.1186/1472-6785-13-30)
Supplement: Additional file 1 — Summary data for variables split by year. [file 1472-6785-13-30-S1.doc]

**Additional file 1.** Summary data for variables split by year

| **Variable** | **2007/08 (n)** | **2008/09 (n)** |
| --- | --- | --- |
| Parasite prevalence (% of infected individuals) | 39.1 (161) | 65.6 (64) |
| Parasite intensity (parasites per 10,000 RBCs) | 0.380 ± 0.175 (44) | NA |
| WBC count (WBCs/RBCs x 1000) | 0.620 ± 0.089 (43) | 0.601 ± 0.062 (40) |
| H:L ratio (heterophils/heterophils + lymphocytes) | 0.293 ± 0.029 (43) | 0.260 ± 0.027 (40) |
| Wing length | 85.855 ± 0.271 (161) | 85.031 ± 0.454 (64) |
| Head-beak length | 30.211 ± 0.051 (161) | 30.142 ± 0.077 (64) |
| Tarsus length | 18.204 ± 0.079 (161) | 18.660 ± 0.213 (64) |
