# Supplementary material for: Avian blood parasite infection during the non-breeding season: an overlooked issue in declining populations?
Source: BMC Ecol. 2013 Sep 6;13:30. doi: 10.1186/1472-6785-13-30 (PMC3848531; doi:10.1186/1472-6785-13-30)
Supplement: Additional file 2 — Results from a GLM to determine whether infection by Haemoproteus, or any interactions therewith, is associated with either tarsus length or head-beak length. For significant terms, parameter estimates with SE are presented (contrasts for Month are against the mean, contrasts for factors with two levels are for the level stated and compared to the other level); for non-significant main effects, statistics are following reinsertion of the term into the minimum adequate model (MAM) and subsequent model comparison. [file 1472-6785-13-30-S2.doc]

**Additional file 2. Results from a GLM to determine whether infection by *Haemoproteus*, or any interactions therewith, is associated with either tarsus length or head-beak length. For significant terms, parameter estimates with SE are presented (contrasts for Month are against the mean, contrasts for factors with two levels are for the level stated and compared to the other level); for non-significant main effects, statistics are following reinsertion of the term into the minimum adequate model (MAM) and subsequent model comparison.**

|  | **Head-beak length** | | | | | **Tarsus length** | | | | |
| --- | --- | --- | --- | --- | --- | --- | --- | --- | --- | --- |
| **Variable** | **df** | **F** | **p** | **Estimate** | **SE** | **df** | **F** | **p** | **Estimate** | **SE** |
| Sex (Male) | 1, 116 | 1.939 | 0.167 | 0.691 | 0.281 | 1 | 0.821 | 0.366 |  |  |
| Age (Juvenile) | 1, 115 | 0.063 | 0.802 | 0.430 | 0.247 | 1, 151 | 6.211 | 0.014 | -0.256 | 0.103 |
| Month (December) | 1, 111 | 2.735 | 0.032 | -0.652 | 0.235 | 1 | 0.383 | 0.821 |  |  |
| Month (January) |  |  |  | -0.038 | 0.196 |  |  |  |  |  |
| Month (February) |  |  |  | -0.081 | 0.138 |  |  |  |  |  |
| Month (March) |  |  |  | 0.670 | 0.183 |  |  |  |  |  |
| Month (April) |  |  |  | 0.101 | 0.162 |  |  |  |  |  |
| Age x Sex | 1, 110 | 5.028 | 0.027 | -0.795 | 0.355 | 1 | 0.61 | 0.43 |  |  |
| Year | 1 | 1.905 | 0.170 |  |  | 1 | 0.026 | 0.870 |  |  |
| *Haemoproteus* infection | 1 | 0.305 | 0.582 |  |  | 1 | 0.682 | 0.410 |  |  |
| *Haemoproteus* infection x Sex | 1 | 0.28 | 0.60 |  |  | 1 | 0.77 | 0.38 |  |  |
| *Haemoproteus* infection x Age | 1 | 0.01 | 0.91 |  |  | 1 | 0.61 | 0.43 |  |  |
| *Haemoproteus* infection x Year | 1 | 0.01 | 0.97 |  |  | 1 | 1.23 | 0.27 |  |  |
